# Noise Cancellation: Viral Fine Tuning of the Cellular Environment for Its Own Genome Replication

**DOI:** 10.1371/journal.ppat.1001158

**Published:** 2010-12-16

**Authors:** Yoshitaka Sato, Tatsuya Tsurumi

**Affiliations:** 1 Division of Virology, Aichi Cancer Center Research Institute, Nagoya, Japan; 2 Department of Virology, Nagoya University Graduate School of Medicine, Nagoya, Japan; 3 Department of Cell Biology, G-COE, Kobe University School of Medicine, Kobe, Japan; 4 Department of Oncology, Graduate School of Pharmaceutical Sciences, Nagoya City University, Nagoya, Japan; University of California San Diego, United States of America

## Abstract

Productive replication of DNA viruses elicits host cell DNA damage responses, which cause both beneficial and detrimental effects on viral replication. In response to the viral productive replication, host cells attempt to attenuate the S-phase cyclin-dependent kinase (CDK) activities to inhibit viral replication. However, accumulating evidence regarding interactions between viral factors and cellular signaling molecules indicate that viruses utilize them and selectively block the downstream signaling pathways that lead to attenuation of the high S-phase CDK activities required for viral replication. In this review, we describe the sophisticated strategy of Epstein-Barr virus to cancel such “noisy” host defense signals in order to hijack the cellular environment.

## Introduction

Cellular DNA damage responses initiate with activation and rapid recruitment of repair proteins to DNA damage sites [1], [2]. Until the damage is repaired, cells are prevented from transitioning to the next stage of the cell cycle. The tumor suppressor p53 is phosphorylated by DNA damage–responsive kinases, resulting in stabilization of p53 and an increase in its protein level. This leads to activation of target gene transcription including p53 itself, which subsequently causes cell cycle arrest or apoptosis [3], [4]. The replicated viral genomes of DNA viruses, including adenoviruses, the polyomavirus, and herpesviruses, are recognized by cellular DNA damage sensors, triggering activation of DNA damage responses [5], [6], [7], [8], [9]. Several lines of evidence revealed viral approaches to create an optimal environment for viral replication by manipulating the host defense systems. In this review, we describe the elegant strategies used by Epstein-Barr virus (EBV) to cancel “noisy” cellular signaling in order to manipulate the cellular environment for its own genome replication.

## Life Cycle of the Epstein-Barr Virus

EBV, a human lymphotropic herpesvirus, infects more than 90% of world's population and is now known to contribute to a variety of human disorders, including infectious mononucleosis, nasopharyngeal carcinoma, Burkitt's lymphoma, and lymphoproliferative diseases occurring in immune-compromised individuals [10]. The lifecycle of EBV is quite distinctive, featuring two alternative infection cycles: “latent” and “lytic.” Primary EBV infection targets resting B lymphocytes, inducing their continuous proliferation. In the resultant B lymphoblastoid cell lines that express a limited number of EBV gene products, the viral genomes are maintained as circular plasmids forming nucleosomal structures with histones [11], and there is no production of virus particles, this being called “latent” infection. In the latent state, viral DNA is replicated only once during S phase, just as host chromosomal DNA [11]. Only a small percentage of infected cells switch their states from the latent stage into the “lytic” cycle to produce progeny viruses. EBV DNA replication occurs at discrete sites in nuclei called “replication compartments,” where all of the viral replication proteins are assembled [12]. During lytic replication, the circular genome becomes a ready template for amplification by the viral replication machinery, generating thousands of copies per cell. This reactivation is correlated with the emergence of human cancers [13], [14]. The switch from latent to lytic replication is triggered by expression of the EBV BZLF1 gene product (also called Zta or ZEBRA) [15]. The BZLF1 protein is a lytic replication origin binding protein and acts to transactivate various viral promoters [16], leading to an ordered cascade of viral gene expression: activation of early genes followed by viral genome replication and late gene expression. Using the EBV system, the alteration in cellular conditions, from latent to virus-productive infection without overlapping signals triggered by virus entry, can be monitored [17], [18].

## Regulation of p53 during the Latent Phase of EBV Infection

**Figure 1:**
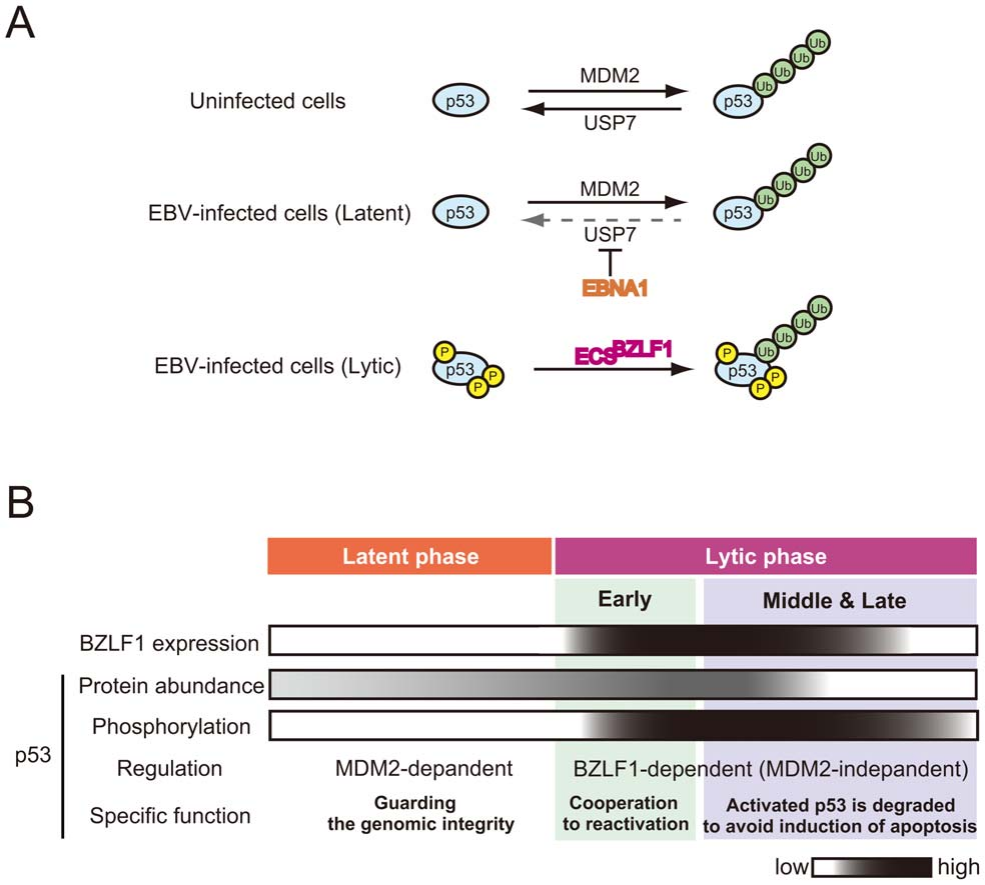
Stage-specific regulation of p53 during EBV infection. (A) The ubiquitination of p53 is regulated by both MDM2 E3 ligase and USP7 deubiquitinase in uninfected cells. During EBV latent infection, EBV latent EBNA1 protein inhibits USP7 and thereby drives the ubiquitination of p53. Phosphorylated p53 is ubiquitinated by BZLF1 protein-associated E3 ligase independently of MDM2 during lytic infection. (B) During the latent phase of EBV infection, p53 is quantitatively regulated by MDM2 ubiquitin ligase via the ubiquitin-proteasome pathway [36], serving as a guardian of genome stability. Expression of BZLF1 protein induces virus-productive (lytic) replication through the ordered cascades of viral gene expression, and concomitantly host DNA damage responses [9], leading to p53 phosphorylation and release of p53 from the MDM2-dependent regulation [36]. In the early stages of lytic infection, the inactive (hypophosphorylated) form of p53 cooperates with viral factors including BZLF1 protein to stimulate virus replication [26], [27]. In the middle and late stages of infection, active (hyperphosphorylated) p53 is ubiquitinated by BZLF1 protein–associated ECS ubiquitin ligase complexes and is degraded in a proteasome-dependent manner to inhibit apoptosis [37].

In uninfected cells, p53 is hypophosphorylated [9] and its level is regulated by cellular E3 ubiquitin ligase MDM2 and cellular ubiquitin–specific protease USP7 (Figure 1A) [19]. The EBV latent protein, EBNA1, contributes to repression of p53-dependent DNA damage signaling by competition of the USP7 binding site with p53 (Figure 1A) [20] (Figure 1A). Furthermore, this interaction between EBNA1 and USP7 leads to the disruption of PML bodies, the nuclear structures important for p53 activation and DNA repair [21]. These findings suggest that EBNA1 expression protects cells from DNA damage–induced apoptosis by destabilizing p53 protein. This possible mechanism points to an important role of EBNA1 in cancer development by allowing uncontrolled cellular proliferation without inducing apoptosis in latent EBV-infected cells.

## The Important Role of p53 in the Early Stages of EBV Lytic Replication

We propose that, during the course of lytic replication, BZLF1 protein plays two distinct roles in the regulation of p53-mediated transactivation, which depend on the progression of lytic replication: the early stage and the middle to late stages (described below) (Figure 1B). Previous studies demonstrated that the EBV immediate-early protein BZLF1, which was either conditionally expressed [22], [23] or overexpressed by a recombinant adenovirus [24], [25], could induce G1 arrest in some cell lines. The BZLF1 protein causes the accumulation of both mRNA and protein of CDK inhibitor p21^Cip1/Waf1^ [23], a well-known p53-target gene product. BZLF1 protein accelerates the rate of p53-DNA complex formation through physical interaction with p53 [26]. In the early phases of lytic replication, p53 is hypophosphorylated and therefore exhibits weak DNA binding ability to its recognition sequences [9]. The BZLF1 protein helps the hypophosphorylated p53 to bind to its recognition sequences, leading to the enhancement of p53-dependent transcription [26]. Levels of p53 and p21^Cip1/Waf1^ are transiently elevated in the early stages of lytic replication, and then decline with the progression of lytic infection [26], probably reflecting the effects of BZLF1 expression.

Recently, we and other groups have shown that p53 is involved in reactivation of EBV [26], [27]. Tsai and his colleagues have reported that induction of viral lytic proteins by a chemical inducer, sodium butylate, does not occur in p53-negative H1299A and Saos2A cells [27], although the ability of BZLF1 or BRLF1 protein to transactivate its downstream genes is not notably affected by the lack of p53 [28], [29]. This implies that p53 might instead be required for a switch from the latent to the lytic cycle. Indeed, we found that overexpression of p53 in the early stages of lytic replication enhances subsequent viral genome replication [26].

In the case of human cytomegalovirus (HCMV), the level of p53 is elevated upon viral infection, but its downstream transcriptional targets remain inactivated [30], [31]. It has been reported that cells infected with HCMV in the absence of p53 produce fewer infectious viral particles and cause delays in viral protein production and trafficking [30]. The HCMV genome has 21 potential p53-responsive sites [32]. HCMV gene expression is thought to be influenced by p53 molecules bound to HCMV genome at immediate-early and early stages of the infection, which could explain the mechanism for reduced and delayed production of virions in p53-negative cells. Similarly, potential p53 recognition sequences are present on the EBV genome (T. Murata et al., unpublished results). Indeed, we have found that p53 is associated with EBV replication compartments [9]. Thus, in the early stages of EBV lytic infection, p53 could be recruited to the EBV genomic regions through its direct binding to the recognition sequences. The BZLF1-mediated enhancement of p53-DNA binding may therefore contribute to the expression of viral genes (Figure 1B).

## Newly Synthesized Viral DNA Elicits Host DNA Damage Responses

**Figure 2:**
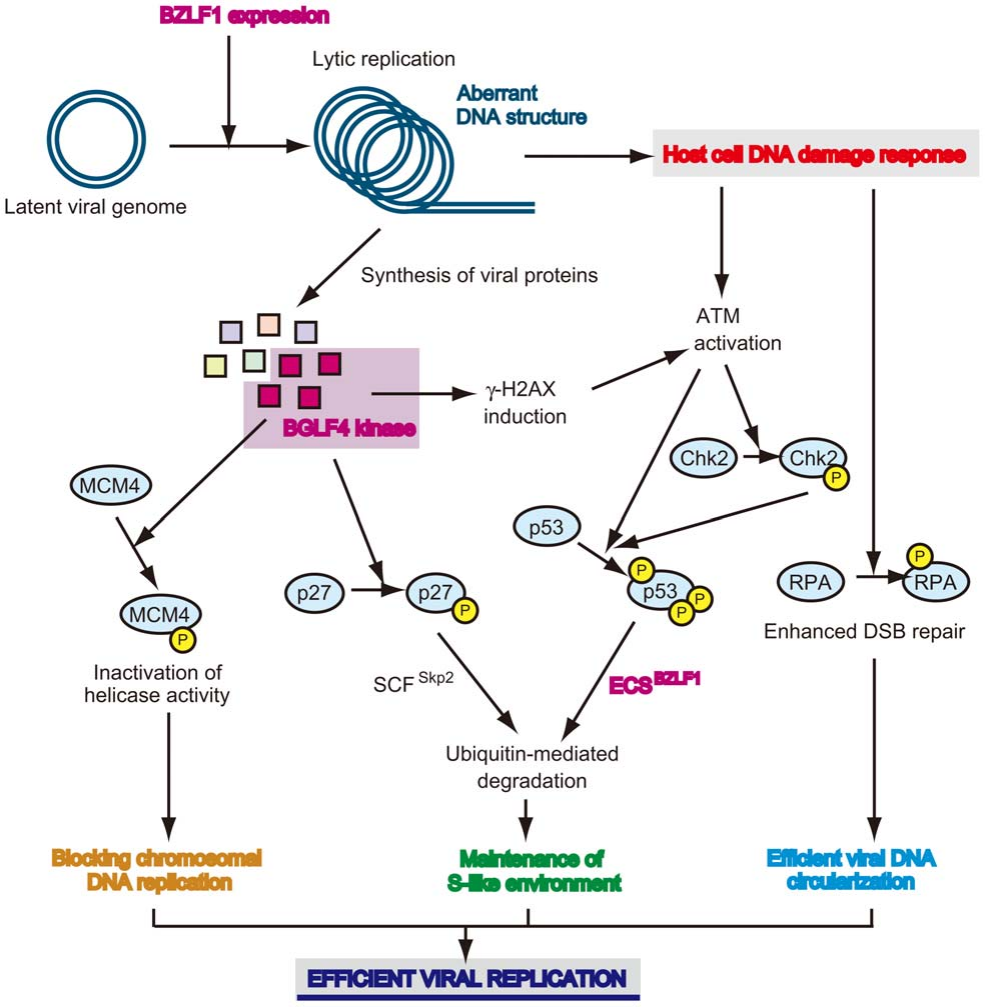
Viral strategy to manipulate the cellular environment for its own genome replication. Induction of lytic replication elicits ATM-dependent host cellular DNA damage responses, because newly synthesized viral DNA is sensed as “aberrant” [9]. The ATM signaling cascade, which is modified by BGLF4 kinase-mediated γ-H2AX induction [35], phosphorylates and activates downstream molecules including CHK2 and p53. However, phosphorylated p53, which can transactivate p21^Cip1/Waf1^ CDK inhibitor, associates with high affinity to BZLF1 protein–formed ECS ubiquitin E3 ligase complex and then is ubiquitinated [37]. On the other hand, EBV protein kinase phosphorylates p27^Kip1^ CDK inhibitor, thereby leading to phosphorylation-mediated ubiquitination by the SCF complex [65]. Since these ubiquitinated proteins are degraded in a proteasome-dependent manner, an S-phase-like environment with high CDK activity required for efficient viral replication is maintained during EBV lytic infection. In parallel with this, replicative helicase activity of the MCM complex is inactivated by BGLF4-mediated phosphorylation of MCM4, causing the inhibition of chromosomal DNA replication [34]. Phosphorylated RPA induced by the DNA damage response stimulates viral DNA replication through homologous recombinational repair [40]. Taken together, EBV manipulates various signaling cascades and thereby achieves efficient viral replication.

Herpesviruses such as HSV, HCMV, and EBV modulate the cell cycle to promote a transition through G1-S phase and achieve the cellular environment with high S-phase CDK activities, called the S-phase-like condition, for virus-productive replication (reviewed in [18]). During the EBV lytic replication, the levels of cyclin E and cyclin A continue to be elevated, and cyclin E- and cyclin A-associated CDK activities actually increase [9]. Moreover, this elevation of S-phase CDK activities drives accumulation of the hyperphosphorylated form of retinoblastoma protein (Rb) and an increase in the level of E2F-1 transcription factor [9]. The observation that chemical CDK inhibitors, such as purvalanol A and roscovitine, block viral lytic replication through prevention of viral immediate-early and early gene expression [33] suggests that a cellular environment featuring high CDK activities is required for efficient viral replication. It is conceivable that expression of proteins involved in DNA metabolism may be promoted under S-phase conditions, when energy generation and other resources support viral replication [18]. However, cellular DNA synthesis is almost entirely blocked during the lytic phase of EBV DNA replication, despite S-phase-like cellular conditions with high CDK activities [17]. The EBV-encoding protein kinase (PK) BGLF4 phosphorylates MCM complex to inhibit its replicative helicase activity (Figure 2) [34]. Although the precise mechanism remains unclear, it might be one of the reasons for inhibition of chromosomal DNA replication and for the blockage of the cell-cycle progression from S to G2 phase.

The host cell DNA damage-sensing machinery recognizes the newly synthesized viral DNA in the lytic phase as “abnormal” DNA, activating ATM-dependent DNA damage signaling [9] (Figure 2). ATM phosphorylates histone H2AX (H2AX), which initiates the DNA damage response. The EBV BGLF4 PK might further amplify this response through phosphorylation of H2AX [35]. ATM phosphorylates p53 at Ser-15, which liberates p53 from MDM2-mediated degradation. The downstream kinases of ATM, Chk1, and Chk2 also phosphorylate p53 at various sites. Therefore, elicitation of DNA damage responses in general activates the transcriptional functions of p53.

## Ubiquitin-Mediated Degradation of p53 in the Middle and Late Stages of Lytic Infection

Paradoxically, reactivation of EBV induces cellular DNA damage responses that causes phosphorylation of p53, which could lead to accumulation of p53 and subsequent activation of p53 downstream signaling (Figure 1), at the same time it establishes the S-phase-like cellular environment. At the middle to late stages of the lytic replication, the p53 target gene products are indeed maintained at low levels [9], [17], [26], [36]. An explanation for this comes from the observation that p53 is degraded via the ubiquitin-proteasome pathway in the middle and late stages of lytic infection, allowing EBV to exploit cellular environments with high CDK activities for efficient viral replication (Figure 2).

A series of recent studies have shown that induction of the EBV lytic program leads to degradation of p53 via a ubiquitin-proteasome pathway independently of MDM2 [36]. The BZLF1 protein functions as an adaptor component of the ECS (Elongin B/C-Cul2/5-SOCS-box protein) ubiquitin ligase complex that targets both unphosphorylated and hyperphosphorylated p53 for degradation (Figure 2) [37]. The BZLF1 M3 mutant, which lacks the ability to bind to Cullin 2 and 5, degrades p53 very little in EBV-positive cells, and the yield of infectious viruses is poorer than in wild-type BZLF1-expressing cells [37]. The BZLF1 M3 mutant includes a mutation at residue E54, previously reported to prevent activation of the EBV lytic cycle, but the underlying mechanism was unknown [38]. These findings suggest that the deficiency of viral replication is due to the failure of p53 degradation.

Chk2 is known to mediate phosphorylation of p53 at Ser-366 and Ser-378 in response to genotoxic stresses [39]. Indeed, p53 is phosphorylated at least at Ser-15, Ser-20, Ser-366, and Ser-378 with progression of EBV lytic infection [37]. Intriguingly, C-terminal phosphorylation of p53 at both Ser-366 and Ser-378 enhances the association with BZLF1 protein and subsequent ubiquitination of p53 [37], possibly through the phosphorylation-induced conformational change of p53. These results suggest that DNA damage responses play a pivotal role in lytic infection.

In addition, inhibition of p53 degradation by the BZLF1 M3 mutant induces apoptotic cellular changes [37]. The maintenance of p53 at very low levels, therefore, is required not only for establishing S-phase-like conditions [9], [17], [36], but also for inhibiting apoptosis for efficient viral propagation. In fact, caspase activity is not induced during lytic infection [40]. Similarly, a body of evidence in the herpesvirus family shows that p53 is inactivated in lytic replication, although its molecular mechanism is controversial [41], [42], [43].

Thus, studies on the relationship between p53 alteration and viral DNA replication have demonstrated that BZLF1 enables hypophosphorylated p53 to transactivate the p53 target genes in the initial phase of lytic replication. In the middle and late stages, activated p53 is subjected to BZLF1-dependent degradation to maintain an S-phase-like environment for efficient viral propagation (Figure 1B).

Several groups have reported that BZLF1 is transiently expressed as an immediate-early gene following EBV primary infection of resting B lymphocytes, although early and late lytic gene expression is very low or undetectable [44], [45], [46]. A transient BZLF1 expression at the primary infection may contribute to establishing a latent infection, as speculated by Wen and colleagues [44]. This could be driven by degradation of p53, which blocks reprogramming of B lymphocyte proliferation. Interestingly, p53 serves as a negative regulator for reprogramming of somatic cells into induced pluripotent stem (iPS) cells [47], [48], [49], [50], [51]. Thus, it is possible that the degradation of p53 by BZLF1 protein-associated ECS ubiquitin ligases contributes to the efficient transformation of B lymphocytes.

## Regulation of CDK Inhibitors during Lytic Replication

The large body of evidence implicating Cullin-based E3 ubiquitin ligase in the regulation of diverse cellular processes [52], [53] provides us with new insights into their significance as potential targets of viruses manipulating the host cellular system. Post-translational modifications, especially phosphorylation and ubiquitination, play a crucial role in cell-cycle progression. Phosphorylation controls the activity of proteins involved in G1-S and G2-M transitions. Ubiquitination and its mediated proteolysis are commonly facilitated to maintain threshold levels of cell-cycle regulators. Two distinct classes of E3 ubiquitin ligase regulate cell-cycle progression [52], possessing an adaptor protein to determine substrate specificity [54], [55], [56]. E3 ligase activity of the anaphase-promoting complex is required for the G2-M transition [57]. The SCF (Skp1-Cul1-F-box protein) family of E3 ligase promotes ubiquitination of phosphorylated substrates and typically targets the mediators of G1-S transition [58]. For instance, ubiquitin-mediated degradation of p27^Kip1^ is regulated by the SCF^Skp2^ complex only when p27^ Kip1^ is phosphorylated at Thr-187 by the cyclin E-CDK2 complex, which induces S phase conditions [59], [60], [61].

The EBV lytic program promotes specific cell cycle–associated activity involved in progression from G1 to S phase, since virus-productive replication occurs under S-phase-like circumstances [18]. Similar to p53, CDK inhibitors are also regulated during lytic replication, contributing to establishment of an S-phase-like cellular environment with high-CDK activities [9], [33]. γ-Herpesviruses possess their own strategies to degrade p27^Kip1^. For example, Kaposi's sarcoma-associated herpesvirus (KSHV)-encoding cyclin (v-cyclin), a latent viral protein, forms a complex with CDK6 to phosphorylate Thr-187 on p27^Kip1^, leading to down-regulation at the protein level [62], [63]. Also, the viral cyclin encoded by murine herpesvirus 68 preferentially associates with CDK2 to phosphorylate Thr-187 on p27^Kip1^ [64]. While EBV does not encode any v-cyclin homologue in its genome, our recent study revealed that the EBV protein kinase BGLF4 can phosphorylate the Thr-187 residue of p27^Kip1^, resulting in its ubiquitination and degradation in an SCF^Skp2^ ubiquitin ligase-dependent manner [65] (Figure 2).

Manipulating the ubiquitin system by EBV involves two aspects of its regulation: attachment of ubiquitin to a substrate and removal from its substrate. As an EBV-encoding deubiquitinating enzyme, BPLF1 deubiquitinates and reduces activity of EBV ribonucleotide reductase [66]. In this case, deubiquitination influences the function of the protein rather than targeting it for proteasomal degradation. A recent paper documented that BPLF1 also act as a deneddylase [67]. Neddylation, which is a conjugation of ubiquitin-like modifier NEDD8 to its substrate, is an important mechanism for regulating Cullin-based E3 ubiquitin ligase [68]. EBV BPLF1 binds to Cullins and attenuates the activity of the Cullin-RING ligases, resulting in accumulation of the licensing factor Cdt1 and induction of DNA re-replication. Inhibition of BPLF1 during the lytic infection prevents viral replication in the cells that carries a recombinant EBV [67]. These findings support the idea that manipulating ubiquitin system by virus promotes viral productive replication. Furthermore, two lytic proteins (BSLF1 and BXLF1) are found as deubiquitinases by a bioinformatic search on the EBV genome [69], although their functions in viral replication remain obscure. Further investigations are needed to determine the exact role of deubiquitination in the context of EBV lytic infection.

The level of another CDK inhibitor protein p21^Cip1/Waf1^, of course, becomes low during lytic replication [36]. Although the detailed mechanisms remain unknown, one reason is that p53 is actively degraded during lytic infection and another is that the SCF^Skp2^ ubiquitin ligase complex directs p21^Cip1/Waf1^ for degradation through S-phase CDK-mediated phosphoryration [70]. Recent study showed that KSHV-encoding microRNA, miR-K1 represses expression of p21^Cip1/Waf^ in latent infection [71]. As an additional mechanism, an EBV-encoding miRNA that has yet to be discovered might regulate p21^Cip1/Waf1^ for maintaining S-phase-like conditions.

On the other hand, maintaining low levels of CDK inhibitors results in accumulation of the hyperphosphorylated Rb protein due to high S-phase CDK activities and causes accumulation of active E2F-1 as lytic replication progresses [9]. E2F-1 in turn activates the transcription of many proteins involved in cellular DNA synthesis and cell-cycle progression [72], and probably transcription of the EBV DNA polymerase gene as well [73]. The available data suggest that E2F activity is required for lytic viral DNA replication. Alternatively, the EBV immediate-early transactivator BZLF1 and BRLF1 proteins are reported to increase the level of E2F-1 [74], [75]. Furthermore, since activated ATM or Chk2 phosphorylates and activates E2F-1 in response to DNA damage [76], [77], the DNA damage response induced by EBV lytic replication could activate E2F-1. To achieve effective viral lytic replication, EBV therefore possesses a variety of strategies to maintain the S-phase-like cellular environment.

## Beneficial Aspects of DNA Damage Signaling on EBV DNA Replication

During EBV lytic replication, phosphorylated ATM and Mre11/Rad50/Nbs1 (MRN) complexes are targeted to replication compartments in nuclei. Simultaneously, homologous recombinational repair (HRR) factors such as replication protein A (RPA), Rad51, and Rad52, as well as MRN complex, are recruited and loaded onto the newly synthesized viral genome in replication compartments [40]. The 32 kDa subunit of RPA is extensively phosphorylated at sites in accordance with these events [40]. Hyperphosphorylation of RPA32 causes a change in RPA conformation, resulting in a switch from catalysis of DNA replication to participation in DNA repair. RNAi knockdown of RPA32 and Rad51 prevents viral DNA synthesis, suggesting that homologous recombination and/or repair of the viral DNA genome might occur, coupled with viral DNA replication to facilitate viral genome synthesis (Figure 2). Thus, the host DNA damage response induced by productive viral replication is essential for efficient EBV lytic genomic replication.

## Conclusions

Replication of DNA viruses in host cells triggers a variety of cellular signaling cascades, including the DNA damage response. Recent studies indicate that such cellular responses to viral genomic replication paradoxically play a crucial role in EBV lytic replication by establishing cellular conditions appropriate for efficient viral replication. To achieve these conditions, EBV manipulates host ubiquitin-proteasome systems, and thereby cancels host antivirus signals. During lytic infection, the interaction between BZLF1 protein and ECS E3 ligase complexes leads to p53 degradation, and the SCF E3 complex recognizes and ubiquitinates phosphorylated p27^Kip1^ through viral protein kinase. Therefore, by skipping the induction of checkpoint signaling and apoptosis, virus-producing cells stay in a persistent S-phase-like environment with high CDK activity.

### Accession numbers

The Entrez Gene (http://www.ncbi.nlm.nih.gov/gene) accession numbers for genes and gene products discussed in this study are as follows: p53 (7157), p21^Cip1/Waf1^ (1026), p27^Kip1^ (1027), USP7 (7874), MDM2 (4193), E2F-1 (1869), ATM (472), Chk2 (11200), H2AX (3014), PARP (142), Skp2 (6502), Cdt1 (81620), ubiquitin (7314), NEDD8 (4738), Rb (5925), Cyclin E (898), Cyclin A (890), CDK2 (1017), CDK6 (1021), RPA32 (6118), Rad51 (5888), Rad52 (5893), KSHV v-cyclin (4961471), and EBV EBNA1 (3783709), BGLF4 (3783704) BPLF1 (3783726), BSLF1 (3783730), BXLF1 (3783741), and BZLF1 (3783744).
